# Editorial: Women in oral epidemiology: 2022

**DOI:** 10.3389/froh.2023.1297040

**Published:** 2023-11-21

**Authors:** D. Roselyn Cerutis, Stefanie Luise Russell, Lucy O'Malley

**Affiliations:** ^1^ Department of Oral Biology and Pharmacology, Creighton University, Omaha, NE, United States; ^2^ Department of Epidemiology and Health Promotion, New York University, New York City, New York, United States; ^3^ Division of Dentistry, School of Medical Sciences, Faculty of Biology, Medicine and Health, The University of Manchester, Manchester, United Kingdom

**Keywords:** oral—general health, oral maternal, oral microbiome, healthcare policy, caries, socioeconomic factors, child oral health, health disparities

**Editorial on the Research Topic** Women in oral epidemiology: 2022

Caries and periodontal diseases are two of the most prevalent non-communicable health issues globally, according to the World Health Organization's 2022 report (1). However, these oral diseases are not evenly distributed across populations, with certain groups bearing a disproportionate burden. People with low socio-economic status (SES) are not only more susceptible to oral health problems but are also more likely to suffer from other concurrent health conditions (2). These articles explore the complex interplay between socio-economic status, oral health, and various factors that impact these dynamics.

While numerous studies have established a connection between SES and oral health, the underlying mechanisms driving this association remain less clear. Oral diseases are typically multifactorial, with genetics and environmental factors interacting to shape an individual’s oral health profile (3). Some research has delved into the intergenerational continuity of oral health, revealing evidence of links between the health of mothers and their young children (4). In this special issue, all articles highlight the interconnectedness of child oral health with their mothers in various ways, such as access to care (Naavaal and Harless), their mothers’ employment status (Awad et al.), or their mothers’ oral microbiome (Li et al.).

Pregnancy introduces significant changes in hormonal balance and the oral microbiome, increasing the risk of developing oral health issues such as gingivitis, periodontal disease and pre-eclampsia, premature birth, and low birth weight; these affect the immediate health of mother and child and predispose them to chronic health problems later in life [reviewed in (5–7)]. The USA is a Republic with 50 states, each responsible for all its public services, including healthcare. Medicaid, a joint federal and state program, covers ∼93.8 m low SES and disabled Americans for dental only until 21 and actual medical coverage varies considerably among states. The Affordable Care Act (“Obamacare”) exacerbated these disparities as only 41 states chose to expand Medicaid eligibility to cover more low-income adults. Naavaal and Harless show it took about four years for statistically significant increases in dental care utilization among pregnant Medicaid-covered women compared to those privately insured, underscoring the importance of early and adequate dental care in promoting overall health and reducing medical care costs for taxpayers.

Access to oral healthcare is influenced by both systemic and individual factors (8) with policy playing a pivotal role in ensuring that suitable services are available (9). Ramphoma et al. propose a groundbreaking policy aimed at addressing the issue of inequitable access to oral healthcare for children and mothers in South Africa. Given its historical context, South Africa grapples with the world’s largest economic disparities among its citizens, with many relying on state-funded healthcare. The Integrated Maternal and Child Oral Health (IMCOH) policy envisages the integration of oral health education, promotion, screening, and referral services into antenatal, perinatal, and neonatal care. Implementing this policy would require effective inter-professional collaboration and has the potential to transform healthcare systems to better meet the needs of populations.

Access to care is one aspect, but prevention of oral diseases hinges largely on behavioral factors (9, 10). Controlling dietary sugar intake is not only beneficial for oral health but also for overall well-being (11). The association between obesity and dental caries, both of which are influenced by sugar consumption, has long intrigued researchers (12). Awad et al. studied caries levels in conjunction with BMI and waist circumference in children residing in the United Arab Emirates, using the employment status of mothers as a proxy measure for SES. This pioneering study found a significant inverse association between waist circumference and caries but no correlation between caries and BMI. While this finding may seem unexpected, it aligns with the existing literature, which is marked by inconsistency. Both obesity and caries are complex conditions, influenced by multiple factors across various levels (13).

Understanding the development of oral diseases in populations necessitates considering multiple factors operating at multiple levels. Microbiota-host interactions have gained recognition as a potent influence on both health and disease as the microbiota are intricately linked with their hosts’ metabolism and overall health [reviewed in (14)]. Li et al. conducted a study investigating the impact of maternal oral microbiota on the microbiome dynamics of healthy infants. The researchers collected whole saliva samples from mothers and infants postpartum, as well as at the 9- and 15-month well-infant visits. Through extensive sequencing and analysis, they identified changes in salivary bacterial species over time. The findings revealed that infants’ oral microbiomes exhibited significant dynamic shifts over time, with increased diversity at 9 and 15 months. This study also monitored the development of oral infections with *S. mutans*. While *S. mutan*s was present in mothers’ saliva at all time-points, only two of 32 children tested positive. These results underscore the need for further research to comprehensively understand the establishment of the early oral microbiome and its implications for lifelong health.

In conclusion, caries and periodontal diseases are widespread global health challenges, with socio-economic status playing a significant role in their prevalence and impact. While the links between SES and oral health are well-established, the precise mechanisms underlying this connection remain complex and multifaceted. Actual healthcare coverage like Medicaid, and policies such as the proposed Integrated Maternal and Child Oral Health policy are vital for addressing disparities in access to care (15). Additionally, understanding oral diseases’ development requires a holistic approach that considers multiple factors at various levels, including the role of microbiota-host interactions. The research articles highlighted in this issue shed light on these intricate dynamics and underscore the importance of increasing comprehensive research efforts for promoting- and achieving- the goal of oral health and overall well-being over the human lifetime.
